# Microfluidic qPCR for detection of 21 common respiratory viruses in children with influenza-like illness

**DOI:** 10.1038/s41598-024-79407-x

**Published:** 2024-11-16

**Authors:** Thomas J. Saville, Hayley Colton, Sheikh Jarju, Edwin P. Armitage, Sainabou Drammeh, Simon Tazzyman, Ya Jankey Jagne, Hadijatou J. Sallah, Elina Senghore, Cariad M. Evans, Thomas C. Darton, Thushan I. de Silva

**Affiliations:** 1 Division of Clinical Medicine, School of Medicine and Population Health, The University of Sheffield, Sheffield, UK; 2 NIHR Sheffield Biomedical Research Centre (BRC) and The Florey Institute of Infection, The University of Sheffield, Sheffield, UK; 3 Department of Virology, Sheffield Teaching Hospitals NHS Foundation Trust, Sheffield, UK; 4 Vaccines and Immunity Theme, Medical Research Council Unit The Gambia at the London, School of Hygiene and Tropical Medicine, PO Box 273, Banjul, The Gambia; 5 Department of Clinical Research, Faculty of Infectious and Tropical Diseases, London School of Hygiene & Tropical Medicine, London, WC1E 7HT UK

**Keywords:** Microfluidics, Polymerase chain reaction, Respiratory virus, Influenza-like illness, Surveillance, Viral infection, Influenza virus, Laboratory techniques and procedures, Epidemiology, Epidemiology, PCR-based techniques, High-throughput screening

## Abstract

Multiple respiratory viruses lead to high morbidity and mortality, yet global surveillance platforms focus primarily on seasonal influenza viruses. The COVID-19 pandemic and new RSV vaccines highlight the importance of a broader approach. Upper respiratory tract swabs from children aged 24–59 months presenting with influenza-like illness in The Gambia were collected during follow-up of a live-attenuated influenza vaccine randomised controlled trial in 2017–18. A microfluidic quantitative polymerase chain reaction (qPCR) assay was established and used to detect 21 respiratory viruses. 76.6% of samples had one or more viruses detected (n = 121/158). The viruses detected most frequently were rhinovirus (n = 37/158, 23.4%) and adenovirus (n = 34/158, 21.5%), followed by parainfluenza virus 3, influenza B and human metapneumovirus B. A third of positive samples had multiple viruses detected (two n = 31/121, 25.6%; three n = 9/121, 7.4%). Our data demonstrates how microfluidic qPCR is a useful tool for high-throughput, comprehensive detection of multiple respiratory viruses in surveillance platforms. Rapidly changing epidemiology exemplifies the need for new, broader approaches to virus surveillance in low-resource settings to respond to future epidemics and to guide the need for and use of new prevention and therapeutic measures.

## Introduction

Viral respiratory tract infections are a common cause of morbidity and mortality worldwide^1,2^. Due to the similar clinical presentation of different respiratory pathogens, molecular testing methods such as quantitative polymerase chain reaction (qPCR) continue to have important diagnostic and epidemiological value^2^.

The COVID-19 pandemic highlighted both the importance of using molecular testing for the surveillance of the underlying aetiology of influenza-like illness (ILI), and the global imbalance of viral surveillance systems. ILI surveillance data from African settings is focused primarily on influenza, and limited data are available for non-influenza respiratory viruses including those with pandemic potential^3–9^.

Multiplex qPCR is widely used to test for multiple respiratory pathogens within a single assay, with the benefit of reducing the total workload and cost compared to multiple singleplex assays^10^. However, the numbers of targets per well must be limited due to potential overlapping of fluorescent groups, and interaction between the primer–probe sets^10^. Microfluidic qPCR may overcome some of these issues by utilising fluidic circuits to channel nanolitres of sample and assay components into multiple single reaction microscopic chambers for amplification and detection. Reported benefits of using microfluidic qPCR over real-time qPCR, include an increased number of targets per run, increased speed of thermal cycling, reduced contamination and substantially reduced sample and reagent volumes^11^. There are, however, few published data available demonstrating the use of microfluidic qPCR for viral detection^12,13^.

Here we used a commercially available microfluidic qPCR system to establish a panel to test for common respiratory viruses in upper respiratory tract (URT) swabs collected from children in The Gambia. Swabs were collected during the passive ILI surveillance followup phase of the NASIMMUNE study, a previously reported randomised control trial live-attenuated influenza vaccine (LAIV)^14^.

## Materials and methods

### Study background

The NASIMMUNE study was a phase 4 randomised controlled trial to primarily explore the mucosal immunological response of children aged 24–59 months to LAIV in The Gambia^14^. Children were eligible if they were clinically well with no history of respiratory illness in the past 14 days. Participants were recruited in two cohorts (30 January to 12 April 2017, and 08 January to 28 March 2018), and followed up until the end of the influenza season of each year, which was predicted to start in mid-June and end in mid-October based on data from Senegal^15,16^. Parents or guardians were reminded at routine study visits (i.e. days 0, 7, 21 and 28 post-LAIV) to bring their children to the study centre if they had symptoms of fever, cough or sore throat. If ILI criteria were met (defined as fever ≥ 37.5 °C or history of fever, plus either a cough or sore throat), an URT swab was collected. The ILI visit ranges of each cohort were 07 February to 31 October in 2017 and 15 January to 07 November in 2018.

### Sample selection and nucleic acid extraction

Viral transport medium (VTM) from URT swabs collected during the NASIMMUNE study were tested where available. Prior to testing, samples were stored at -80 °C without any known freeze thaw cycles. Nucleic acids were extracted manually from samples using the Applied Biosystems MagMAX™ Viral/Pathogen Nucleic Acid Isolation Kit (A42352) according to the manufacturer’s protocol. 400 µl of sample was extracted, eluted into a final volume of 50 µl using nuclease-free water and stored at -80 °C.

### Microfluidic qPCR assay

The respiratory virus primer–probe sets used in the microfluidic qPCR assay were adapted from the United Kingdom Accreditation Service (UKAS)-accredited PCR assay used at the Sheffield Teaching Hospitals NHS Foundation Trust (STH) Laboratory Medicine department for routine laboratory detection of respiratory viruses from clinical samples. These primer–probe sets were based on prior published sequences of adenovirus, human metapneumovirus (HMPV) A, HMPV-B, influenza A, influenza B, parainfluenza virus 1, parainfluenza virus 2, parainfluenza virus 3, parainfluenza virus 4, rhinovirus, RSV-A, RSV-B and seasonal coronaviruses (229E, NL63,OC43)^17^. We updated the sets for influenza A and influenza B in line with World Health Organisation (WHO) protocols, and RSV-A and RSV-B to more recently published sequences^18,19^. Additional primer–probe sets for seasonal coronavirus HKU1, human bocavirus 1, human bocaviruses 2–4 and severe acute respiratory syndrome coronavirus-2 (SARS-CoV-2) RdRP, E and N were added (Supplementary Table S1).

Positive assay controls consisted of plasmids containing an amplified region of each target (Eurofins Scientific and Integrated DNA Technologies (IDT)) (Supplementary Table S2). Targets were split across two plasmids to reduce plasmid complexity. The final working stock was supplemented with separate SARS-CoV-2 and RNase P (an internal control to indicate extraction failure) plasmid controls obtained from IDT, as the manufacturers could not include these in the custom plasmids. Each run had an extraction blank of nuclease-free water taken from extraction through the entire process, plus a negative control of nuclease-free water was added at the reverse transcription reaction.

The Juno™ and Biomark™ systems from Standard BioTools™ (formerly Fluidigm) are commercially available instruments used in conjunction to perform microfluidic qPCR. Microfluidic qPCR is performed on an Integrated Fluidics Circuit (IFC), which can be ordered in a range of sizes. We utilised the 192.24 Dynamic Array™ IFC for Gene Expression which has 192 sample wells and 24 assay wells, facilitating 4,608 individual qPCR reactions on a single IFC.

Extracted samples underwent reverse transcription (RT), and complementary DNA (cDNA) was synthesised by incubating 4 µl of LunaScript® RT SuperMix (M3010L) with 16 µl of extract (25 °C for 2 min, 55 °C for 10 min, 95 °C for 1 min). A pre-amplification 0.2✕ assay pool mix was made up from the forward and reverse primers of each qPCR target at a concentration of 180 nM. Of this 0.2✕ assay pool mix, 3.125 µl was taken forward and added to 6.25 µl of cDNA from the reverse transcription step, plus 2.5 µl Fluidigm Preamp Master Mix (100–5581), and 0.625 µl nuclease-free water. The pre-amplification reaction was incubated at 95 °C for 2 min, followed by 20 thermal cycles of 95 °C for 15 s, and 60 °C for 4 min. The pre-amplified product was diluted with 50 µl of Invitrogen™ TE Buffer (12,090,015)^20,21^.

**Fig. 1:**
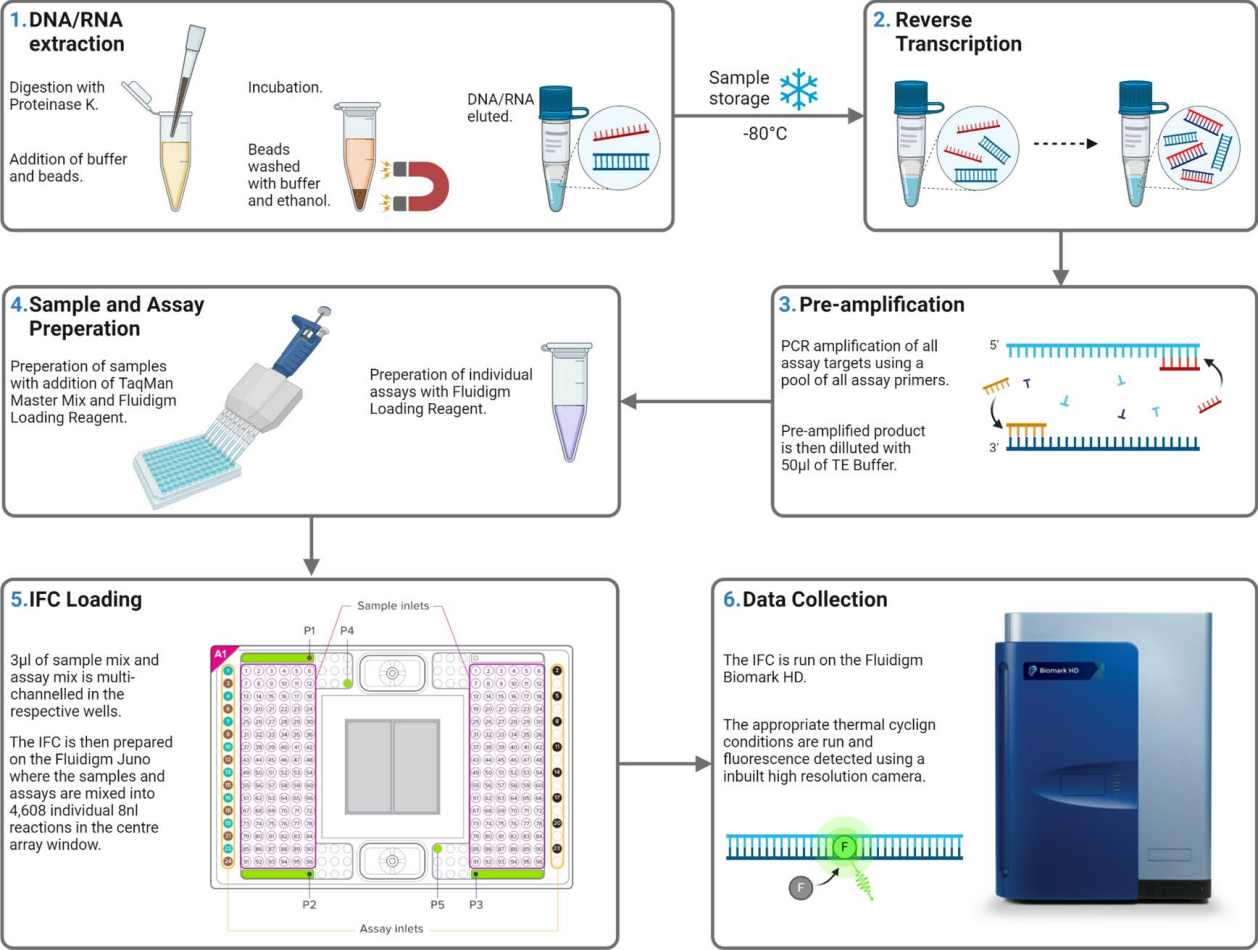
Schematic of sample processing and microfluidic assay. Created with BioRender.com.

Prior to qPCR, 1.8 µl of the diluted pre-amplified product was mixed with 2 µl of Applied Biosystems™ TaqMan™ Fast Advanced Master Mix (4,444,557) and 0.2 µl of Fluidigm 20X GE Sample Loading Reagent (100–7610). This was loaded into individual sample wells on a Fluidigm 192.24 Dynamic Array™ IFC for Gene Expression (GE). Primer–probe sets were added to the assay wells and primed on the Juno™, which mixed nanolitre volumes of the components for each qPCR reaction into the central PCR chambers of the IFC. The IFC was run using the GE 192 × 24 Fast v1 protocol (95 °C for 60 s, followed by 35 cycles: 96 °C for 5 s, 60 °C for 20 s) on the Biomark™ instrument^20^ (Fig. 1).

Samples were processed and tested in duplicate following the reverse transcription stage to ensure replication of results. Therefore, per 192.24 IFC use we were able to test 93 samples (including the extraction blanks), plus the two positive controls (10,000 and 1,000 copies/µl) and the negative control. Two quantification cycle (Cq) values per sample were available for analysis, and samples were deemed positive if either Cq value was below the threshold (Table 1). If the initial results were indeterminate, or if there was a failure of any of the controls, the extract was retested provided there was sufficient volume remaining.

**Table 1:** Limit of detection assay results (copies/μl and Cq values) and STH sample microfluidic results.

Viral Target	LOD, copies/μl	Corresponding Cq value	Positive STH samples under LOD Cq (%)	Negative STH samples over LOD Cq (%)	Adjusted Cq value
SARS-CoV-2 (RdRP)	6.25	25.721	16/16 (**100%**)	26/26 (**100%**)	25.310^d^
SARS-CoV-2 (E)	9.66	19.886	18/19 (**94.7%**)	26/26 (**100%**)	17.230^d^
SARS-CoV-2 (N)	< 6.25	-	0^b^ ( −)	26/26 (**100%**)	18.010^d^
Influenza A	42.4	21.907	5/6 (**83%**)	60/60 (**100%**)	–
Influenza B	42.4	24.305	0^a^ ( −)	66/66 (**100%**)	–
Human bocavirus 1	42.4	22.241	0^b^ ( −)	0^b^ ( −)	–
Human bocavirus 2–4	23.5	25.276	1^b^ ( −)	0^b^ ( −)	–
RSV-A	40.4	22.075	10/11 (**90.9%**)^c^	55/55 (**100%**)	–
RSV-B	34.5	24.606		55/55 (**100%**)	–
Rhinovirus	36.9	22.588	13/23 (**56.5%**)	42/42 (**100%**)	26.500^e^
HMPV-A	44.9	23.173	0^a^	66/66 (**100%**)	–
HMPV-B	25.9	24.113	0^a^	65/66 (**98.5%**)	–
Adenovirus	36.2	21.810	4/8 (**50%**)	57/61 (**93.4%**)	25.440^e^
Parainfluenza virus 1	34.5	21.290	0	66/66 (**100%**)	–
Parainfluenza virus 2	34.5	21.355	1/1 (**100%**)	65/65 (**100%**)	–
Parainfluenza virus 3	36.7	26.521	0^a^ ( −)	66/66 (**100%**)	–
Parainfluenza virus 4	34.5	21.110	0^a^ ( −)	66/66 (**100%**)	–
Seasonal coronavirus 229E	34.5	21.151	0^a^ ( −)	63/63 (**100%**)	–
Seasonal coronavirus OC43	36.7	19.528	0^a^ ( −)	63/63 (**100%**)	–
Seasonal coronavirus NL63	34.5	20.159	2/2 (**100%**)	62/63 (**98.4%**)	–
Seasonal coronavirus HKU1	42.4	17.156	0^b^ ( −)	0^b^ ( −)	–

No positive samples from STH available.

*Cq* quantification cycle, *HMPV* Human Metapneumovirus, *LOD* Limit of Detection, *SARS-CoV-2* Severe Acute Respiratory Syndrome Coronavirus-2, *STH* Sheffield Teaching Hospitals, *RdRP* RNA-Dependant RNA Polymerase, *RNA* Ribonucleic acid, *ROC* Receiver operating characteristic, *RSV* Respiratory Syncytial Virus.

^a^No positive samples from STH available.

^b^Not in STH panel.

^c^RSV-A n=5/10, RSV-B n=4/10.

^d^Modified following ROC analysis.

^e^Modified following comparative testing.

### Assay validation

A limit of detection (LOD) assay was performed whereby the positive control plasmids (Supplementary Table S2) were tested in replicates of 20, at two fold dilutions (200, 100, 50, 25, 12.5, and 6.25 copies/μl). The LOD for each target was calculated using a standard replicate curve of positive control material, with a nonlinear least squares model to calculate the corresponding Cq value from the lowest concentration at which 95% of replicates were positive (Table 1 and Supplementary Figure S1)^22^. We compared our LOD values with those previously reported in the primer/probe source publications if available (supplementary Table S3).

To validate the LOD Cq thresholds, we obtained fully anonymised residual nucleic acid extracts from URT swabs stored in viral transport media (VTM) from Sheffield Teaching Hospitals NHS Foundation Trust (STH) in the United Kingdom. All residual extracts were collected in August 2022 and were stored at -80 °C following respiratory virus PCR testing as part of routine clinical care. During clinical diagnostics, the samples were extracted and then tested for SARS-CoV-2 and/or respiratory virus PCR depending on the clinical indication (Supplementary Table S4).

SARS CoV-2 targets RdRP, E and N showed particularly low LOD and so receiver-operator characteristic (ROC) curves were plotted to further investigate the target’s sensitivity and specificity (Supplementary Figure S2). As N gene testing was not used in STH at the time, N gene grouping was based on E gene positivity.

### Statistical analysis

**Fig. 2:**
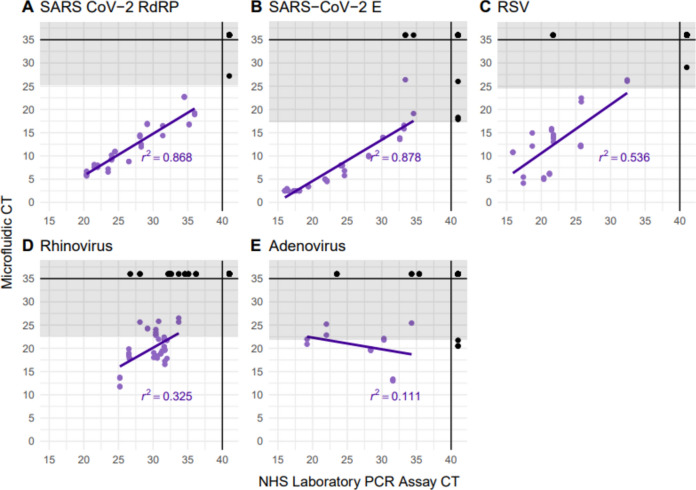
Correlation between microfluidic and NHS Laboratory PCR Cq values. The grey shaded areas represent the Cq values over the 95% LOD threshold. Datapoints are coloured based on if Cq values are available on both assays (purple), or if there was “No Cq” on at least one of the assays (black). Correlation was calculated including only the purple points. *Cq* Quantification cycle, *PCR* Polymerase Chain Reaction, *RdRP* RNA-Dependant RNA Polymerase, *RNA* Ribonucleic acid, *RSV* Respiratory Syncytial Virus, *SARS-CoV-2* Severe Acute Respiratory Syndrome Coronavirus-2.

qPCR data including the amplification curves were processed and reviewed using Standard BioTools Biomark and EP1 real-time PCR analysis software. Analysis and production of figures were performed using R version 4.3.1. ROC curves were plotted to compare the microfluidic qPCR Cq values of known positive and negative samples using the one-way analysis functions in GraphPad Prism 9.3.1 (Supplementary Figure S2). For viruses where eight or more known positive samples were available, correlation between STH and microfluidic PCR testing was assessed by fitting a linear regression model to calculate the r^2^ value (Fig. 2).

### Cost

Costings for reagents and consumables were calculated per sample in January 2024 and compared for three qPCR setups: QuantStudio singleplex qPCR (£105.23), QuantStudio 4 × multiplex (i.e. four primer–probe sets per well) qPCR (£26.56) and Standard BioTools Microfluidic GE IFC 192.24 qPCR (£24.14) (Supplementary Material Costings.xlsx). Sample collection and extraction were excluded as this would be the same for all methods. Hands-on lab work time was not costed but estimated at 22 days, 5.5 days and 1 day respectively. Processing cost and time were calculated as manual labour (i.e. without additional robotic automation) to allow for direct comparison.

### Ethics approval statement

Ethical approval for the NASIMMUNE study (NCT02972957) was provided by The Gambia Government/Medical Research Council Joint Ethics Committee (SCC1555) and the Medicines Control Agency of The Gambia. The study was done according to the International Conference on Harmonisation Good Clinical Practice standards. Anonymised residual nucleic acid extracts from STH patients taken during routine clinical care were used for assay validation with approval from the STH Research & Development office.

### Patient consent statement

A parent or guardian provided written or thumb printed informed consent for their child or children to participate. If parents/guardians were not English literate, an impartial witness was present throughout the informed consent discussion undertaken in a local language, and signed to confirm completeness of the consent provided.

## Results

### Assay validation

78 residual nucleic acid extracts were available from STH for assay validation. From the STH PCR results, 65 had a positive result for at least one virus (n = 65/78, 83.3%), with five of these testing positive for two viruses. The positive results included rhinovirus (n = 23), SARS-CoV-2 (n = 19), RSV (n = 11), adenovirus (n = 8), influenza A (n = 6), seasonal coronavirus (n = 2) and parainfluenza 2 (n = 1). Using the LOD thresholds, results were reported positive on microfluidic testing if either of the two duplicate Cq values were below the threshold defined in Table 1. From the known positive STH samples, we found that most targets were resulted appropriately on microfluidic testing (Table 1).

Based on the SARS CoV-2 ROC curves, the microfluidic Cq values with optimal sensitivity and specificity were 25.310, 17.230 and 18.010 for RdRP, E and N targets respectively (Supplementary Figure S2 and Supplementary Table S5).

The STH and microfluidic PCR scatter plots (Fig. 2) show Cq values seen in microfluidic testing were lower than when compared to the STH Cq values. The highest r^2^ value was seen with SARS-CoV-2 E gene (0.878), and the lowest with adenovirus (-0.111). This was speculated to be due to STH performing adenovirus PCR as a singleplex, with PCR conditions tailored specifically to the adenovirus primers and probes. Additional consistency statistical analysis for STH and microfluidic PCR results was performed to test the reliability of the results. Cronbach’s Alpha values of 0.950, 0.922, 0.890, and 0.744 for SARS CoV-2 RdRP, SARS CoV-2 E, RSV, and Rhinovirus respectively indicate high reliability. The Cronbach’s Alpha value of 0.582 for Adenovirus was the lowest, although within an acceptable level of reliability.

As many of the known rhinovirus and adenovirus positive samples had Cq values on microfluidic testing above the previously defined cut offs (based on 95% LOD), we opted to increase the Cq values to 26.5 and 25.4 respectively, in order to capture all of the known positives. The final adjusted Cq thresholds used are shown in Table 1.

### Application of microfluidic qPCR assay to the detection of respiratory viruses in children with influenza-like illness

365 children were recruited (n = 178 in 2017, and n = 187 in 2018), of which 330 completed the study (lost to follow up after day 21 post-vaccination n = 23, withdrew consent n = 12). 27.0% of children presenting to an unscheduled visit with any of the ILI symptoms (fever, cough or sore throat), met the criteria for ILI (n = 166/614). VTM from 158 URT swabs collected at ILI visits were available (n = 84 in 2017 and n = 74 in 2018), representing 110 participants attending between 1–4 times over the follow up period. Samples positive for influenza A or influenza B that were collected within 21 days of LAIV administration were presumed to be detection of vaccine strain rather than infection, and were therefore excluded from the analysis for influenza (n = 4/158, 2.5%).

**Fig. 3:**
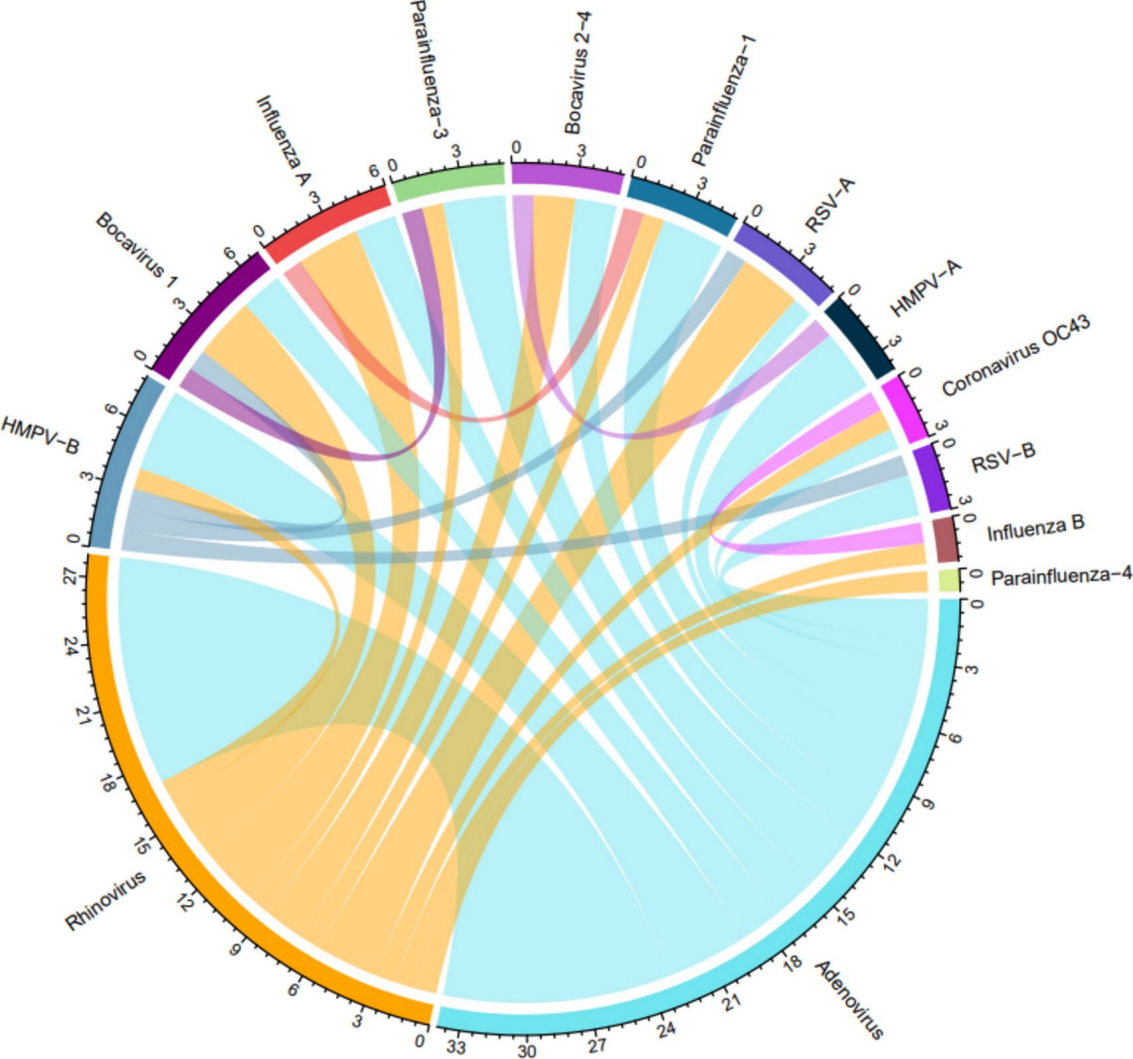
Chord diagram showing pairs of respiratory viruses detected in co-infections (i.e. 2 or more viruses per swab) of children with ILI detected by passive surveillance in the Gambia. Colours correspond to the seasonality data shown in Fig. 4. Where more than two (one pair of) viruses were detected, each infection may be represented more than once. *ILI* Influenza-like illness, *HMPV* Human metapneumovirus, *RSV* Respiratory syncytial virus.

Most samples had at least one virus detected (n = 121/158, 76.6%). Rhinovirus and adenovirus were the most frequently detected viruses in both years (Table 2). All other viruses were detected at varying frequency except SARS-CoV-2, parainfluenza virus 2 and seasonal coronavirus 229E (Table 2). Cq value distributions can be seen in Supplementary Figure S3. Almost a third of the positive samples had multiple viruses detected (two viruses n = 31/121, 25.6%; three viruses n = 9/121, 7.4%). The most common co-infection was between the two most commonly detected viruses rhinovirus and adenovirus (n = 11/40, 27.5%) (Fig. 3), however the viruses detected in isolation the least often were human bocaviruses 2–4 (n = 0/3, 0.0%), adenovirus (n = 7/34, 20.6%) and human bocavirus 1 (n = 2/7, 28.6%) (Table 2).

**Table 2:** Respiratory viruses detected in influenza-like illness episodes in children aged 24–59 months in The Gambia in 2017 and 2018.

Virus	2017, n	2018, n	Number of samples with virus detected, n (% total)	Mono-infection frequency (%)
Rhinovirus	23	14	37 (23.4)	15/37 (40.5)
Adenovirus	17	17	34 (21.5)	7/34 (20.6)
Parainfluenza 3	10	4	14 (8.8)	10/14 (71.4)
Influenza B	9	3	12 (7.6)	10/12 (83.3)
Human metapneumovirus B	7	5	12 (7.6)	6/12 (50.0)
Influenza A	5	5	10 (6.3)	6/10 (60.0)
RSV-A	3	6	9 (5.7)	5/9 (55.6)
Parainfluenza 1	1	8	9 (5.7)	5/9 (55.6)
Human metapneumovirus A	7	0	7 (4.4)	4/7 (57.1)
Human Bocavirus 1	5	2	7 (4.4)	2/7 (28.6)
Seasonal coronavirus OC43	5	1	6 (3.8)	3/6 (50.0)
RSV-B	5	0	5 (3.2)	3/5 (60.0)
Human Bocavirus 2–4	1	2	3 (1.9)	0/3 (0.0)
Parainfluenza 4	3	0	3 (1.9)	2/3 (66.7)
Seasonal coronavirus NL63	0	2	2 (1.3)	2/2 (100.0)
Seasonal coronavirus HKU1	0	2	2 (1.3)	2/2 (100.0)
Parainfluenza 2	0	0	0	–
Seasonal coronavirus 229E	0	0	0	–
SARS-CoV-2	0	0	0	–

Respiratory viruses are ordered from highest to lowest prevalence.

*RSV* Respiratory Syncytial Virus, *SARS-CoV-2* Severe Acute Respiratory Syndrome Coronavirus-2.

**Fig. 4:**
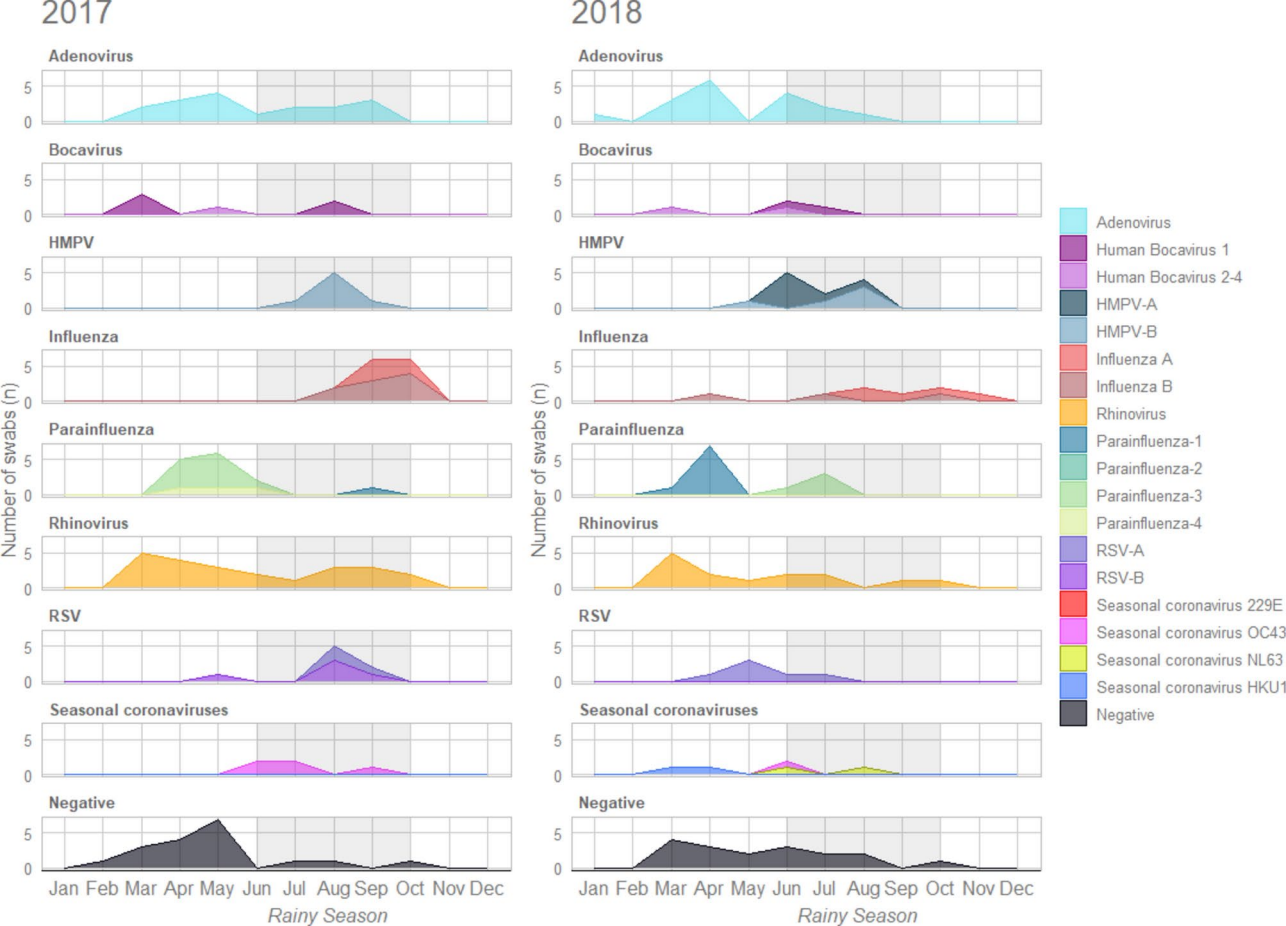
Seasonal detection of respiratory viruses from upper respiratory tract swabs taken from children aged 24–59 months with influenza-like illness in the Gambia in 2017 and 2018. Rainy season months are shaded in grey. Only partial data are available for January and November, and no data for December, due to follow up windows in the parent study. *HMPV* Human Metapneumovirus, *RSV* Respiratory Syncytial Virus.

The number of children presenting with ILI for each respiratory virus detected was analysed per month (Fig. 4). Two peaks in attendance for ILI were seen over the course of follow up, one prior to the rainy season and the second during the rainy season (Supplementary Figure S4). We found that the peak months of adenovirus (April–May) and rhinovirus (March) detection was consistent across both years. HMPV-A and influenza viruses were most frequently detected during the rainy season in both years, however detection of influenza B in 2017 was higher than in 2018. The other viruses did not appear to have a consistent seasonal pattern. A larger proportion of swabs taken earlier in the follow up period in March–May had no respiratory virus detected (14.3–31.8%) compared to later on in the year including the rainy season (0.0–25.0%) (Fig. 4, Supplementary Figure S4.

Symptom data were available for most participants with a positive sample (n = 115/121, 95.0%) (Supplementary Table S6). Children with RSV (n = 4/8, 50.0%) or influenza (n = 9/18, 50.0%) were more frequently febrile at their ILI visit than those testing positive for other viruses (8.6–18.1%). The presence of cough, sore throat, or history of fever in participants were similar between the different viruses, although it was noted that sore throat was infrequently reported (0.0–3.0%).

## Discussion

In our cohort of young children presenting with ILI in the Gambia, we successfully applied a microfluidic qPCR technique using targets for multiple respiratory viruses. Using this assay has revealed a number of interesting and noteworthy findings, including the high frequency of adenovirus in addition to rhinovirus infection, and the high detection rate of multiple viruses in upper respiratory tract swabs of children with ILI.

Whilst previous studies have shown that rhinovirus incidence in West Africa is high, adenovirus is typically detected less frequently^5,23,24^. However, in some studies of children with ILI or serious illness, adenovirus has been reported as frequently as influenza and RSV^5,23,25^. In a study of children under 15 years hospitalised with ILI in Cameroon, the most common virus identified was adenovirus (27.3%), which was detected throughout the year with little temporal change^26^. Interestingly, recent seroprevalence data from West Africa has demonstrated a very high seroprevalence of human adenovirus (HAdV) IgG in children under 5 in Nigeria (98.5%), and a large proportion of adults in Sierra Leone had detectable neutralising antibodies towards more pathogenic adenoviruses such as HAdV-7 (65.7%)^27,28^. Further investigation into circulating strains of adenovirus in West Africa is indicated, both to investigate ILI aetiology, and also to ensure minimal pre-existing adenoviral immunity from previous infection for the development of effective adenoviral vectored vaccines^29^.

Co-infections with multiple viruses were common in our cohort of children presenting with ILI. Although bocaviruses are frequently detected alongside other viruses and are not routinely included in many respiratory viral panels, a retrospective study of children in the UK found that up to a third of bocavirus mono-infection cases required intensive care^30^. The value of expanding surveillance to a broader range of respiratory viruses was also demonstrated in a large study in Belgium with virus-associated severe acute respiratory infection (SARI), where co-infections with multiple non-influenza respiratory viruses were found to be almost as frequent as influenza mono-infection in children under 5^31^. We also found a similar proportion of children who had no virus detected as this study, which could suggest either that other potential viral targets should be assessed (e.g. influenza C), and/or other illnesses (both infective and non-infective) are an underlying cause of ILI presentation^31^.

A robust global surveillance programme for the detection of a broad range of respiratory viruses in patients presenting with ILI/SARI is paramount for pandemic preparedness, plus the increasing number of available preventative and therapeutic interventions for respiratory viruses, such as maternal vaccines and long acting monoclonal antibodies for RSV^6,32–34^. Current surveillance of respiratory viruses within Africa is primarily for influenza detection in sentinel countries reporting to the Global Influenza Surveillance and Response System (GISRS)^3,6,8,9,15^. Existing influenza surveillance platforms have successfully been used to identify and test for Middle East Respiratory Syndrome coronavirus (MERS-CoV) and SARS-CoV-2, demonstrating that existing infrastructure is adaptable to include other viruses^6^. While newer molecular methods including microfluidic qPCR assays could significantly strengthen existing surveillance systems, the current WHO guidance on the molecular detection of respiratory viruses addresses each virus individually^18,35^. Thus, a more unified approach would be required for this broader method of surveillance.

To be utilised as a surveillance assay, the running costs of such high throughput assays need to be considered, particularly for use in low to middle income countries (LMIC). When compared to a 96-well manual singleplex qPCR setup, a manual 4 × multiplex setup reduces the cost per sample from £105.23 down to £26.56. The use of the microfluidic qPCR setup we used (£24.14) is similar to manual 4 × multiplexing, and reduces hands-on lab work from ~ 5.5 days to ~ 1 day. Other factors would have to be considered depending on the setting, such as start up and utility costs, plus the availability of reagents. Situating multiplex diagnostics for ILI in the appropriate level of health care or surveillance setting would depend on the policy prioritisation for disease prevention and ability to fund these additional costs required.

A particular advantage of using microfluidic qPCR over multiplex qPCR is that primer–probe sets remain in singleplex, thus issues with primer–probe cross reactivity are avoided. It is also straightforward to switch out primer–probe sets to respond rapidly to evolving epidemics and emerging viruses with pandemic potential^12,36^. The option to multiplex with a microfluidic qPCR setup is possible if there are more targets of interest, although care must be taken to optimise primer–probe sets and fluorophores within each reaction and further validation would need to take place.

Our study has some limitations, including that samples were not collected from participants between the two cohorts (late October 2017 to mid January 2018). As samples were taken post-LAIV, we recognise that influenza rates may be lower than if unvaccinated children were studied. Additionally some primer–probe sets need further validation, either as positive samples were not available from the period of collection, or testing was not available locally at Sheffield Teaching Hospitals Laboratory Medicine department for comparison (e.g. bocaviruses 1–4 and seasonal coronavirus HKU1). Defining the Cq cut off for each target using a microfluidic qPCR assay needs careful consideration, as the ideal cycle limit to be used for microfluidic detection with a pre-amplification step is assay dependent. Typical real-time PCR assays are often limited to 40 cycles, as this theoretically allows a single target molecule to be amplified to over a trillion copies, with any reactivity over 40 cycles deemed to be due to amplification or artefact^37^. Standard BioTools’ Biomark protocol recommends 35 thermal cycles, plus a LOD experiment to establish the Cq cut off for each target^20^. For example, their Advanta Dx SARS-CoV-2 RT-PCR assay uses a Cq threshold of 32 (6.25 genome equivalents/μL) following a 95% LOD experiment, with a reported 100% sensitivity and specificity^38^. However we found the Cq values generated from our LOD assay required adjustment following further testing of known positive and negative samples.

## Conclusion

Microfluidic qPCR assays show potential for high throughput and cost-effective molecular detection of multiple respiratory viruses. They could be a useful tool to broaden current surveillance and can be rapidly adapted to test for emerging viruses. Our data show the high frequency and diversity of viruses causing ILI in children in The Gambia, and exemplifies the need for new, more comprehensive approaches to virus surveillance in LMIC to respond to future epidemics and to guide the need for and use of new prevention and therapeutic measures. Further investigation into circulating strains of adenovirus in West Africa may be indicated, for surveillance and vaccine development purposes.

## Supplementary Information

Supplementary Information 1.

Supplementary Information 2.

## Data Availability

The datasets generated during and/or analysed during the current study are available from the corresponding authors on reasonable request.
