# Supplementary material for: Microfluidic qPCR for detection of 21 common respiratory viruses in children with influenza-like illness
Source: Sci Rep. 2024 Nov 16;14:28292. doi: 10.1038/s41598-024-79407-x (PMC11569225; doi:10.1038/s41598-024-79407-x)
Supplement: Supplementary file 1 — Supplementary Information 1. [file 41598_2024_79407_MOESM1_ESM.pdf]

Microfluidic qPCR for Detection of 21 Common Respiratory Viruses in Children with Influenza-like Illness

Notes

To compare: qPCR - single-plex, qPCR - multiplex, Fluidigm 192.24
1 run = 96 samples in duplicate and 22 targets
Calculate cost per 96 sample run and per sample
Largest bulk options selected for pricing across everything
All proccess are costed to be performed manually i.e. without robotic automation.
January 2024

**Run definition**	**1 run**
All samples (inc. blanks and controls)	96
Patient samples	93
Targets	22
Replicates	2
Multiplexing	4

				Cost per sample	£105.23		Cost per sample	£26.56		Cost per sample	£24.14	
				Days work	22		Days work	5.5		Days work	1	
				**qPCR simgleplex**	**£9,786.34**		**qPCR multiplex**	**£2,469.67**		**Fluidigm**	**£2,245.35**	
**Product**	**Product ID**	**Product uses**	**Product price**	**Uses per run**	**Cost per run**	**Notes**	**Uses per run**	**Cost per run**	**Notes**	**Uses per run**	**Cost per run**	**Notes**
NEB LunaScript® RT SuperMix Kit	E3010L	100	£471.00	1056	£4,973.76		264	£1,243.44		96	£452.16	4ul per sample
Azenta Life Sciences FrameStar 96 Well Skirted Low Profile PCR Plates - Clear Wells/Clear Frame	4ti-0960/C	50	£148.56	22	£65.37		6	£17.83		4	£11.88	1xRT + 2xPreAmp + 1xPreLoading
Applied Biosystems™ MicroAmp™ Optical 96-Well Reaction Plate	4316813	500	£2,310.00	44	£203.28		11	£50.82		0	£0.00	
Applied Biosystems™ TaqMan™ Fast Advanced Master Mix for qPCR	4444558	5000	£3,390.00	4224	£2,863.87		1056	£715.97		38.4	£26.04	2ul used instead of 10ul so divided by 5
Starlab 10/20 µl XL graduated TipOne® Filter Tip (sterile)	S1120-3810-C	80	£757.93	154	£1,459.02		39	£369.49		10	£94.74	as boxes of 96 tips
Starlab 20 µl UltraPoint® graduated TipOne® Filter Tip, Natural, (sterile)	S1123-1810-C	80	£744.30	22	£204.68		6	£55.82		3	£27.91	as boxes of 96 tips
Starlab 200 µl graduated TipOne® Filter Tip, Natural (sterile)	S1120-8810-C	80	£744.30	1	£9.30		1	£9.30		2	£18.61	as boxes of 96 tips
Standard Biotools Preamp Master Mix—5 Tubes	100-5581	200	£1,200.00	0	£0.00		0	£0.00		192	£1,152.00	Increased PreAmp usage results in 200 reactions from this product instead of 500 as stated by the manufacturer
Standard Biotools 192.24 GE IFC & 2X Reagent Kit - 50 IFCs	102-0583	50	£23,100.00	0	£0.00		0	£0.00		1	£462.00	
IDT 250nmole DNA Oligo X39	-	31590000	£1,365.00	7488	£0.32		7488	£0.32		8.58	£0.00	X39 ~450ul of 100uM
TaqMan custom MGB 6nmol probes X10	4316034	1080000	£3,480.00	1920	£6.19		1920	£6.19		2.2	£0.01	X10 60ul of 100uM
IDT 100nm PrimerTime 5' 6-FAM/ZEN/3'IBFQ custom probes X10	-	7200000	£920.00	1920	£0.25		1920	£0.25		2.2	£0.00	X10 ~400ul of 100uM
IDT SARS-CoV-2 (E) reverse primer IDT product E Sarbeco R2 Reverse Primer, 100 nmol	10006891	1800000	£205.00	192	£0.02		192	£0.02		0.22	£0.00	1000ul of 100uM
IDT SARS-CoV-2 (E) probe IDT product E Sarbeco P1 (FAM) Probe, 50 nmol	10006893	900000	£380.00	192	£0.08		192	£0.08		0.22	£0.00	500ul of 100uM
IDT RNase P forward primer IDT product RNase P Forward Primer Aliquot, 100 nmol	10006836	1800000	£205.00	192	£0.02		192	£0.02		0.22	£0.00	1000ul of 100uM
IDT RNase P reverse primer IDT product RNase P Reverse Primer Aliquot, 100 nmol	10006837	1800000	£205.00	192	£0.02		192	£0.02		0.22	£0.00	1000ul of 100uM
IDT RNase P probe IDT product RNase P (FAM) Probe Aliquot, 50 nmol	10006838	900000	£380.00	192	£0.08		192	£0.08		0.22	£0.00	500ul of 100uM
Eurofins Standard Gene - Viral Multiplex Control 1	-	1300000	£231.12	44	£0.01		11	£0.00		1	£0.00	
IDT Gene 501-1500 bp -Viral Multiplex Control 2	-	980000	£123.00	44	£0.01		11	£0.00		1	£0.00	
IDT 2019-nCoV N Positive Control	10006625	50000	£68.00	44	£0.06		11	£0.01		1	£0.00	
IDT Hs RPP30 Positive Control	10006626	50000	£56.00	44	£0.05		11	£0.01		1	£0.00	
IDT 2019-nCoV E Positive Control	10006896	50000	£69.00	44	£0.06		11	£0.02		1	£0.00	
