# Supplementary material for: Microfluidic qPCR for detection of 21 common respiratory viruses in children with influenza-like illness
Source: Sci Rep. 2024 Nov 16;14:28292. doi: 10.1038/s41598-024-79407-x (PMC11569225; doi:10.1038/s41598-024-79407-x)
Supplement: Supplementary file 2 — Supplementary Information 2. [file 41598_2024_79407_MOESM2_ESM.pdf]

Microfluidic qPCR for Detection of 21 Common Respiratory Viruses in Children with Influenza-like Illness

Supplementary Material

Target	Primers and probe sequence	Probe label	Amplified sequence
SARS-CoV-2 (RdRP)[1]	Forward<br>GTGARATGGTCATGTGTGGCGG<br>Reverse<br>CARATGTTAAASACACTATTAGCATA<br>Probe<br>CAGGTGGAACCTCATCAGGAGATGC	5′6-FAM/ZEN/3′IBFQ	GTGAAATGGTCATGTGTGGCGGTTCACTATATGTTAAACCAGGTGGAACCTCATCAGGAGATGCCACAACTGCTTATGCTAATAGTGTTTTTAACATTTG
SARS-CoV-2 (E)[1]	Forward<br>ACAGGTACGTTAATAGTTAATAGCGT<br>Reverse*<br>ATATTGCAGCAGTACGCACACA<br>Probe*<br>ACACTAGCCATCCTTACTGCGCTTCG	5′6-FAM/ZEN/3′IBFQ	ACAGGTACGTTAATAGTTAATAGCGTACTTCTTTTTCTTGCTTTCGTGGTATTCTTGCTAGTTACACTAGCCATCCTTACTGCGCTTCGATTGTGTGCGTACTGCTGCAATAT
SARS-CoV-2 (N)[1]	Forward<br>CACATTGGCACCCGCAATC<br>Reverse<br>GAGGAACGAGAAGAGGCTTG<br>Probe<br>ACTTCCTCAAGGAACAACATTGCCA	5′6-FAM/ZEN/3′IBFQ	CACATTGGCACCCGCAATCCTGCTAACAATGCTGCAATCGTGCTACAACTTCCTCAAGGAACAACATTGCCAAAAGGCTTCTACGCAGAAGGGAGCAGAGGCGGCAGTCAAGCCTCTTCTCGTTCCTC
Influenza A (Matrix)[2]	Forward<br>CCMAGGTCGAAACGTAYGTTCTCTCTATC<br>Reverse<br>TGACAGRATYGGTCTTGTCTTTAGCCAYTCCA<br>Probe<br>CAGGCCCCCTCAAAGCCGARAT	5′6-FAM/3′MGBNFQ	CCGAGGTCGAAACGTATGTTCTCTCTATCGTTCCATCAGGCCCCCTCAAAGCCGAGATCGCGCAGAGACTTGAAGATGTCTTTGCTGGGAAAAACACAGATCTTGAGGCTCTCATGGAATGGCTAAAGACAAGACCAATTCTGTCA
Influenza B (NS)[2,3]	Forward<br>GGAGCAACCAATGCCAC<br>Reverse<br>GTKTAGGCGGTCTTGACCAG<br>Probe<br>ATAAACTTYGAAGCAGGAAT	5′6-FAM/3′MGBNFQ	GGAGCAACCAATGCCACCATAAACTTCGAAGCAGGAATTCTGGAGTGCTATGAAAGACTTTCATGGCAAAGGGCCCTTGACTACCCTGGTCAAGACCGTCTAAAC
Human bocavirus 1 (NS1)[4]	Forward<br>CTGCTGCACTTCCTGATTCAAT<br>Reverse<br>GGAGCTTCTTCCAGAGATGTTC<br>Probe<br>ACTGCATCCGGTCTC	5′6-FAM/3′MGBNFQ	CTGCTGCACTTCCTGATTCAATCAGACTGCATCCGGTCTCCGGCGAGTGAACATCTCTGGAAGAAGCTCC
Human bocavirus 2-4 (NS1)[5]	Forward<br>ATGCACTTCCGCATYTCGTC<br>Reverse<br>GGAGCTCTTYCCAGAGATGTTC<br>Probe<br>ACTGCATCCGGTCTC	5′6-FAM/3′MGBNFQ	ATGCACTTCCGCATCTCGTCAGACTGCATCCGGTCTCCGGCGAGTGAACATCTCTGGGAAGAGCTCC
Respiratory syncytial virus A (L)[6]	Forward<br>AATACAGCAAAATCYAACCAACTTTAYA<br>Reverse<br>GCCAAGGAAGCATGCARTARA<br>Probe<br>CYTTARTRCACAATAGCA	5′6-FAM/3′MGBNFQ	AATACAGCAAAATCCAACCAACTTTACACTACTACTTCCCACCAAATATCCTTAGTGCACAATAGCACATCACTTTACTGCATGCTTCCTTGGC

Respiratory syncytial virus B (L)[6]	Forward<br>AATACAGCAAAATCYAACCAACTTTAYA<br>Reverse<br>GCCAAGGAAGCATGCARTARA<br>Probe<br>GACATCYTTAGTAAGGAAYAGTG	5’6-FAM/3’MGBNFQ	AATACAGCAAAATCTAACCAA<br>CTTTACATCACCACTTCACATC<br>AGACATCTTTAGTAAGGAATA<br>GTGCATCACTTTATTGCATGCT<br>TCCTTGGC
Rhinovirus (5’UTR)[7]	Forward<br>TGGACAGGGTGTGAAGAGC<br>Reverse<br>CAAAGTAGTCGGTCCCATCC<br>Probe<br>TCCTCCGGCCCCTGAATG	5’6-FAM/ZEN/3’IBFQ	TGGACAGGGTGTGAAGAGCCT<br>ACTATTGCGCTTAGTTGTGAGT<br>CCTCCGGCCCCTGAATGCGGC<br>TAATCCCTAAACCCCGGTACGC<br>CTCGTGGTGTAAACCAACATTT<br>GCAAGGTCGTAATGAGTAATT<br>CTGGGATGGGACCGACTACTT<br>TG
Human metapneumovirus A (Fusion)[7]	Forward<br>GCYGTYAGCTTCAGTCAATTCAA<br>Reverse<br>TCCAGCATTGTCTGAAAATTGC<br>Probe<br>AGAAGGTTTCTAAATGTTG	5’6-FAM/3’MGBNFQ	GCCGTTAGCTTCAGTCAATTCA<br>ACAGAAGGTTTCTAAATGTTGT<br>GCGGCAATTTTCAGACAATGC<br>TGGA
Human metapneumovirus B (Fusion)[7]	Forward<br>GCYGTYAGCTTCAGTCAATTCAA<br>Reverse<br>GTTATCCCTGCATTGTCTGAAAACT<br>Probe<br>AGAAGATTCCTAAATGTTGTGCG	5’6-FAM/3’MGBNFQ	GCCGTTAGCTTCAGTCAATTCA<br>ACAGAAGATTCCTAAATGTTGT<br>GCGGCAGTTTTCAGACAATGC<br>AGGGATAAC
Adenovirus (Matrix)[7]	Forward<br>GCCCCAGTGGTCTTACATGCACATC<br>Reverse<br>GCCACGGTGGGGTTTCTAAACTT<br>Probe<br>TCGGAGTACCTGAGCCCGGGTCTGGTGCA	5’6-FAM/ZEN/3’IBFQ	GCCCCAGTGGTCTTACATGCAC<br>ATCTCGGGCCAGGACGCCTCG<br>GAGTACCTGAGCCCGGGTCTG<br>GTGCAGTTTGCCCGCGCCACC<br>GAGACGTACTTCAGCCTGAAT<br>AACAAGTTTAGAAACCCCACC<br>GTGGC
Parainfluenza virus 1 (HN)[7]	Forward<br>GATTTAAACCCGGTAATTTCTCA<br>Reverse<br>CCTTGTTCCTGCAGCTATTACAGA<br>Probe<br>ACCTATGACATCAACGAC	5’6-FAM/3’MGBNFQ	GATTTAAACCCGGTAATTTCTC<br>ATACCTATGACATCAACGACAA<br>CAGGAGATCATGTTCTGTAAT<br>AGCTGCAGGAACAAGG
Parainfluenza virus 2 (HN)[7]	Forward<br>ATGAAAACCATTTACCTAAGTGATGGA<br>Reverse<br>CCTCCYGGTATRGCAGTGACTGAAC<br>Probe<br>TCAATCGCAAAAGC	5’6-FAM/3’MGBNFQ	ATGAAAACCATTTACCTAAGTG<br>ATGGAATCAATCGCAAAAGCT<br>GTTCAGTCACTGCTATACCAGG<br>AGG
Parainfluenza virus 3 (HN)[7]	Forward<br>CCAGGGATATAYTAYAAAGGCAAAA<br>Reverse<br>CCGGGRCACCCAGTTGTG<br>Probe<br>TTTCTCGGRTATGGAGGTCTTGAACAYCCA	5’6-FAM/ZEN/3’IBFQ	CCAGGGATATACTACAAAGGC<br>AAAATAATATTTCTCGGGTATG<br>GAGGTCTTGAACATCCAATAA<br>ATGAGAATGCAATCTGCAACA<br>CAACTGGGTGTCCCGG
Parainfluenza virus 4 (Fusion)[7]	Forward<br>CAGAYAACATCAATCGCCTTACAAA<br>Reverse<br>TGTACCTATGACTGCCCCAAARA<br>Probe<br>CCMATCACAAGCTCAGAAATYCAAAGTCGT	5’6-FAM/ZEN/3’IBFQ	CAGACAACATCAATCGCCTTAC<br>AAAGCCAATCACAAGCTCAGA<br>AATTCAAAGTCGTTTCTTTGGG<br>GCAGTCATAGGTACA

Human coronavirus 229E (Nucleocapsid)[7]	Forward<br>CAGTCAAATGGGCTGATGCA<br>Reverse<br>AAAGGGCTATAAAGAGAATAAGGTATTCT<br>Probe<br>TGAACCACAACGTGGTCGTCAGGG	5'6-FAM/ZEN/3'IBFQ	CAGTCAAATGGGCTGATGCAT<br>CTGAACCACAACGTGGTCGTC<br>AGGGTAGAATACCTTATTCTCT<br>TTATAGCCCTTT
Human coronavirus OC43 (Nucleocapsid)[7]	Forward<br>CGATGAGGCTATTCCGACTAGGT<br>Reverse<br>CCTTCCTGAGCCTTCAATATAGTAACC<br>Probe<br>TCCGCCTGGCACGGTACTCCCT	5'6-FAM/ZEN/3'IBFQ	CGATGAGGCTATTCCGACTAG<br>GTTTCCGCCTGGCACGGTACTC<br>CCTCAGGGTTACTATATTGAAG<br>GCTCAGGAAGG
Human coronavirus NL63 (1a gene)[7]	Forward<br>ACGTACTTCTATTATGAAGCATGATATTAA<br>Reverse<br>AGCAGATCTAATGTTATACTTAAAACTACG<br>Probe<br>ATTGCCAAGGCTCCTAAACGTACAGGTGTT	5'6-FAM/ZEN/3'IBFQ	ACGTACTTCTATTATGAAGCAT<br>GATATTAAAGTTATTGCCAAG<br>GCTCCTAAACGTACAGGTGTT<br>ATTTTGACACGTAGTTTTAAGT<br>ATAACATTAGATCTGCT
Human coronavirus HKU1 (Nucleocapsid)[8]	Forward<br>GATCCTACTAYTCAAGAAGCTATCCCTACT<br>Reverse<br>ACCTTCCTGAGCCTTCAACATAAT<br>Probe<br>TTTYCGCCTGGTACGATTTTGCCTC	5'6-FAM/ZEN/3'IBFQ	GATCCTACTACTCAAGAAGCTA<br>TCCCTACTAGGTTTCCGCCTGG<br>TACGATTTTGCCTCAAGGCTAT<br>TATGTTGAAGGCTCAGGAAGG<br>T
RNase P[9]	Forward*<br>AGATTTGGACCTGCGAGCG<br>Reverse*<br>GAGCGGCTGTCTCCACAAGT<br>Probe*<br>TTCTGACCTGAAGGCTCTGCGCG	5'6-FAM/ZEN/3'IBFQ	AGATTTGGACCTGCGAGCGGG<br>TTCTGACCTGAAGGCTCTGCGC<br>GGACTTGTGGAGACAGCCGCT<br>C

Supplementary Table S1: Primer/probe sets. All HPLC purified.
Green = Forward Primer, Purple = Probe, Red = Reverse Primer
*IDT catalogue products

- SARS-CoV-2 (E) reverse primer IDT product E_Sarbeco_R2 Reverse Primer, 100 nmol, 10006891
- SARS-CoV-2 (E) probe IDT product E_Sarbeco_P1 (FAM) Probe, 50 nmol, 10006893
- RNase P forward primer IDT product RNase P Forward Primer Aliquot, 100 nmol, 10006836
- RNase P reverse primer IDT product RNase P Reverse Primer Aliquot, 100 nmol, 10006837
- RNase P probe IDT product RNase P (FAM) Probe Aliquot, 50 nmol, 10006838

1. **SARS-CoV-2 RdRP, E and N:** Corman VM, Landt O, Kaiser M, Molenkamp R, Meijer A, Chu DK, et al. Detection of 2019 novel coronavirus (2019-nCoV) by real-time RT-PCR. Eurosurveillance. 2020 Jan 23;25(3).
2. **Influenza A and B:** WHO information for the molecular detection of influenza viruses [Internet]. 2021. Available from: https://cdn.who.int/media/docs/default-source/influenza/molecular-detention-of-influenza-viruses/protocols_influenza_virus_detection_feb_2021.pdf?sfvrsn=df7d268a_5
3. **Influenza B:** Nakauchi M, Takayama I, Takahashi H, Oba K, Kubo H, Kaida A, et al. Real-time RT-PCR assays for discriminating influenza B virus Yamagata and Victoria lineages. Journal of Virological Methods. 2014 Sep;205:110–5.
4. **Human Bocavirus 1:** Ligozzi M, Diani E, Lissandrini F, Mainardi R, Gibellini D. Assessment of NS1 gene-specific real time quantitative TaqMan PCR for the detection of Human Bocavirus in respiratory samples. Molecular and Cellular Probes. 2017 Aug;34:53–5.
5. **Human Bocavirus 2-4:** Ligozzi M, Diani E, Lissandrini F, Mainardi R, Gibellini D. Assessment of NS1 gene-specific real time quantitative TaqMan PCR for the detection of Human Bocavirus in respiratory samples. Molecular and Cellular Probes. 2017 Aug;34:53–5.
6. **Respiratory Syncytial Virus A and B:** Todd AK, Costa AM, Waller G, Daley AJ, Barr IG, Deng YM. Rapid detection of human respiratory syncytial virus A and B by duplex real-time RT-PCR. Journal of Virological Methods. 2021 Aug;294:114171.
7. **Rhinovirus, Human metapneumovirus A and B, Adenovirus, Parainfluenza virus 1, 2, 3, and 4, and Human Coronavirus 229E, OC43, and NL63:** Gunson RN, Carman WF. During the summer 2009 outbreak of "swine flu" in

Scotland what respiratory pathogens were diagnosed as H1N1/2009?. BMC Infectious Diseases. 2011 Jul 13;11(1).

8. **Human Coronavirus HKU1:** Hasan MR, Al Mana H, Young V, Tang P, Thomas E, Tan R, et al. A novel real-time PCR assay panel for detection of common respiratory pathogens in a convenient, strip-tube array format. Journal of Virological Methods. 2019 Mar;265:42–8.
9. **RNase P:** Emery SL, Erdman DD, Bowen MD, Newton BR, Winchell JM, Meyer RF, et al. Real-Time Reverse Transcription–Polymerase Chain Reaction Assay for SARS-associated Coronavirus. Emerging Infectious Diseases [Internet]. 2004 Feb 1;10(2):311–6. Available from: https://www.ncbi.nlm.nih.gov/pmc/articles/PMC3322901/

Control Name (Supplier)	Amplified Sequences	Length	Sequence
Viral Multiplex Control 1<br><br>(Eurofins Scientific)	Influenza A,<br>Influenza B,<br>Seasonal coronavirus HKU1,<br>Human bocavirus 1,<br>Respiratory syncytial virus A,<br>Rhinovirus,<br>Human metapneumovirus A,<br>Adenovirus	856 bp	CCGAGGTCGAAACGTATGTTCTCTCTATCGTTCCATCAGGCCCC<br>CTCAAAGCCGAGATCGCGCAGAGACTTGAAGATGTCTTTGCTG<br>GGAAAAACACAGATCTTGAGGCTCTCATGGAATGGCTAAAGA<br>CAAGACCAATTCTGTCAGGAGCAACCAATGCCACCATAAACTT<br>CGAAGCAGGAATTCTGGAGTGCTATGAAAGACTTTCATGGCA<br>AAGGGCCCTTGACTACCCTGGTCAAGACCGTCTAAACGATCCT<br>ACTACTCAAGAAGCTATCCCTACTAGGTTTCCGCCTGGTACGAT<br>TTTGCCTCAAGGCTATTATGTTGAAGGCTCAGGAAGGTCTGCT<br>GCACTTCCTGATTCAATCAGACTGCATCCGGTCTCCGGCGAGT<br>GAACATCTCTGGAAGAAGCTCCAATACAGCAAAATCCAACCAA<br>CTTTACACTACTACTTCCCACCAAATATCCTTAGTGCACAATAG<br>CACATCACTTTACTGCATGCTTCCTTGGCTGGACAGGGTGTGA<br>AGAGCCTACTATTGCGCTTAGTTGTGAGTCCTCCGGCCCCTGA<br>ATGCGGCTAATCCCTAAACCCCGGTACGCCTCGTGGTGTAAAC<br>CAACATTTGCAAGGTCGTAATGAGTAATTCTGGGATGGGACCG<br>ACTACTTTGGCCGTTAGCTTCAGTCAATTCAACAGAAGGTTTCT<br>AAATGTTGTGCGGCAATTTTCAGACAATGCTGGAGCCCCAGTG<br>GTCTTACATGCACATCTCGGGCCAGGACGCCTCGGAGTACCTG<br>AGCCCGGGTCTGGTGCAGTTTGCCCGCGCCACCGAGACGTACT<br>TCAGCCTGAATAACAAGTTTAGAAACCCCACCGTGGC
Viral Multiplex Control 2<br><br>(IDT)	Parainfluenza virus 1,<br>Parainfluenza virus 2,<br>Parainfluenza virus 3,<br>Parainfluenza virus 4,<br>Seasonal coronavirus 229E,<br>Seasonal coronavirus OC43,<br>Seasonal coronavirus NL63,<br>Human bocavirus 2-4,<br>Human metapneumovirus B,<br>Respiratory syncytial virus B	820 bp	GATTTAAACCCGGTAATTTCTCATACCTATGACATCAACGACAA<br>CAGGAGATCATGTTCTGTAATAGCTGCAGGAACAAGGATGAA<br>AACCATTTACCTAAGTGATGGAATCAATCGCAAAAGCTGTTCA<br>GTCACTGCTATACCAGGAGGCCAGGGATATACTACAAAGGCA<br>AAATAATATTTCTCGGGTATGGAGGTCTTGAACATCCAATAAA<br>TGAGAATGCAATCTGCAACACAACTGGGTGTCCCGGCAGACA<br>ACATCAATCGCCTTACAAAGCCAATCACAAGCTCAGAAATTCA<br>AAGTCGTTTCTTTGGGGCAGTCATAGGTACACAGTCAAATGGG<br>CTGATGCATCTGAACCACAACGTGGTCGTCAGGGTAGAATACC<br>TTATTCTCTTTATAGCCCTTTCGATGAGGCTATTCCGACTAGGT<br>TTCCGCCTGGCACGGTACTCCCTCAGGGTTACTATATTGAAGG<br>CTCAGGAAGGACGTACTTCTATTATGAAGCATGATATTAAAGT<br>TATTGCCAAGGCTCCTAAACGTACAGGTGTTATTTTGACACGTA<br>GTTTTAAGTATAACATTAGATCTGCTATGCACTTCCGCATCTCG<br>TCAGACTGCATCCGGTCTCCGGCGAGTGAACATCTCTGGGAAG<br>AGCTCCGCCGTTAGCTTCAGTCAATTCAACAGAAGATTCCTAA<br>ATGTTGTGCGGCAGTTTTCAGACAATGCAGGGATAACAATACA<br>GCAAAATCTAACCAACTTTACATCACCACTTCACATCAGACATC<br>TTTAGTAAGGAATAGTGCATCACTTTATTGCATGCTTCCTTGGC

Supplementary Table S2: Details and Sequences of custom positive control plasmids

Green = Forward Primer, Purple = Probe, Red = Reverse Primer

IDT commercially available supplementary controls:

- 2019-nCoV_N_Positive Control, 10006625
  nCoV-N control: Severe acute respiratory syndrome coronavirus 2 isolate Wuhan-Hu-1, complete genome (GenBank: NC_045512.2)
- Hs_RPP30 Positive Control, 10006626
- 2019-nCoV_E Positive Control, 10006896
  2019-nCoV_E control: Severe acute respiratory syndrome coronavirus 2 isolate Wuhan-Hu-1, complete genome (GenBank: NC_045512.2)

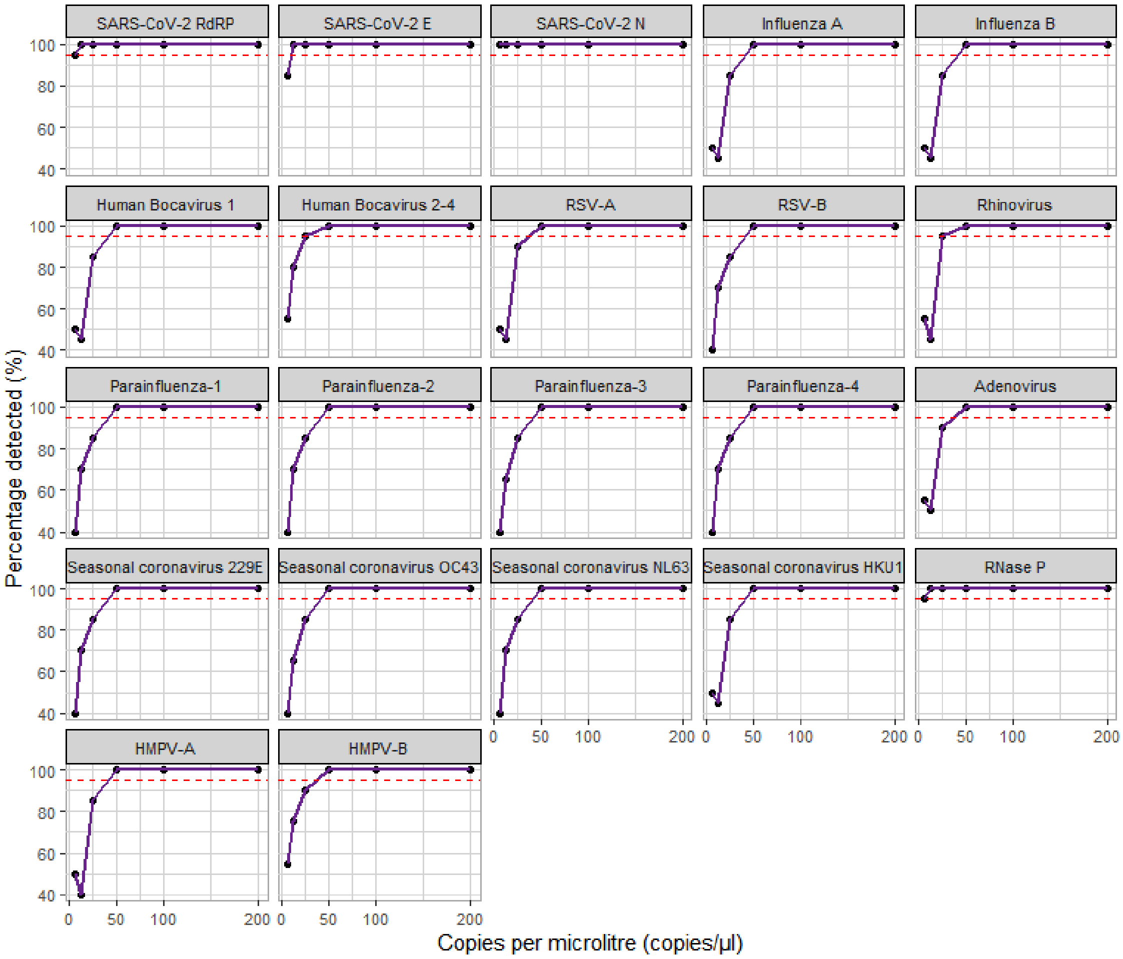

Supplementary Figure S1: 95% limit of detection (LOD) calculation of positive control plasmids.
The LOD of the assay was calculated using the intersection between the red dashed line (95% of 20 replicates testing positive on qPCR) and the purple line (standard replicate curve based on experiment results). The LOD of SARS-CoV-2 N gene target could not be determined as all replicates were positive at the final dilution of 6.25 copies/μl.
HMPV: Human Metapneumovirus, SARS-CoV-2: Severe Acute Respiratory Syndrome Coronavirus-2, RdRP:RNA-Dependant RNA Polymerase, RNA: Ribonucleic acid, RNaseP: Ribonuclease P, RSV: Respiratory Syncytial Virus

Viral Target	Microfluidic LOD, copies/µl	Previous reported standard qPCR LOD copies/µl
SARS-CoV-2 (RdRP)	6.25	0.72
SARS-CoV-2 (E)	9.66	0.78
SARS-CoV-2 (N)	<6.25	1.66
Influenza A	42.4	1.50
Influenza B	42.4	1.65
Human bocavirus 1	42.4	1.50
Human bocavirus 2-4	23.5	1.50
RSV-A	40.4	50.00
RSV-B	34.5	200.00
Rhinovirus	36.9	Not reported
HMPV-A	44.9	Not reported
HMPV-B	25.9	Not reported
Adenovirus	36.2	Not reported
Parainfluenza virus 1	34.5	Not reported
Parainfluenza virus 2	34.5	Not reported
Parainfluenza virus 3	36.7	Not reported
Parainfluenza virus 4	34.5	Not reported
Seasonal coronavirus 229E	34.5	Not reported
Seasonal coronavirus OC43	36.7	Not reported
Seasonal coronavirus NL63	34.5	Not reported
Seasonal coronavirus HKU1	42.4	Not reported

Supplementary Table S3: Comparison of Standard Biotools Microfluidic assay Limit of Detection (LOD) with previously reported LOD for each target.

	Assays in use	Targets	Information available
**Standard respiratory virus testing**[1,2]	UKAS accredited in-house multiplex	Influenza A<br>Influenza B<br>Parainfluenza virus 1<br>Parainfluenza virus 2<br>Parainfluenza virus 3<br>Parainfluenza virus 4<br>Respiratory syncytial virus (A+B)<br>Human metapneumovirus (A+B)<br>Rhinovirus<br>Seasonal coronaviruses (OC43, NL63, 229E)<br>Adenovirus	Cq value for each target
**SARS-CoV-2**[2]	a) In-house PCR assay[3]	E gene<br>RdRP gene	Cq value for each gene
	b) Hologic Aptima™NAAT	Two conserved regions of ORF1 ab gene	RLU value

Supplementary Table S4: Summary of STH Respiratory Viral molecular testing

CT: Cycle threshold, NAAT: Nucleic Acid Amplification Test, PCR: Polymerase chain reaction, RLU: Relative light unit, UKAS: United Kingdom Accreditation Service

1. The previously described primers and probes are split into a combination of single-well singleplex and multiplex assays. The targets for RSV (A, B), HMPV (A, B), and seasonal coronaviruses (229E, OC43, NL63) were pooled, so the specific causative species within these viruses is not specified
2. Samples are extracted on the automated MagNA Pure 96 extraction system (Roche) prior to testing
3. Colton H, Ankcorn M, Yavuz M, Tovey L, Cope A, Raza M, Keeley AJ, State A, Poller B, Parker M, de Silva TI, Evans C. Improved sensitivity using a dual target, E and RdRp assay for the diagnosis of SARS-CoV-2 infection: Experience at a large NHS Foundation Trust in the UK. J Infect. 2021 Jan;82(1):159-198. doi: 10.1016/j.jinf.2020.05.061. Epub 2020 May 28. PMID: 32474037; PMCID: PMC7255707.

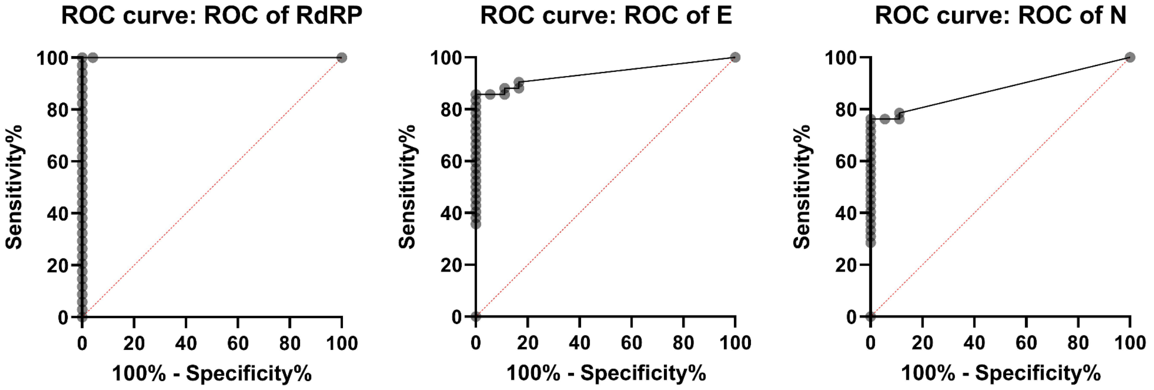

Supplementary Figure S2: ROC curves for the SARS-CoV-2 targets: RdRP, E and N genes.

RdRP:RNA-Dependant RNA Polymerase, RNA: Ribonucleic acid, ROC: Receiver operating characteristic

SARS-CoV-2 RdRP					
CT	Sensitivity %	95% CI	Specificity %	95% CI	Likelihood ratio
< 23.32	97.06	85.08% to 99.85%	100	86.20% to 100.0%	
< 25.31	100	89.85% to 100.0%	100	86.20% to 100.0%	
< 31.61	100	89.85% to 100.0%	95.83	79.76% to 99.79%	24

SARS-CoV-2 E					
CT	Sensitivity %	95% CI	Specificity %	95% CI	Likelihood ratio
< 16.21	83.33	69.40% to 91.68%	100	82.41% to 100.0%	
< 17.23	85.71	72.16% to 93.28%	100	82.41% to 100.0%	
< 18.08	85.71	72.16% to 93.28%	94.44	74.24% to 99.72%	15.43
< 18.71	85.71	72.16% to 93.28%	88.89	67.20% to 98.03%	7.714
< 22.58	88.1	75.00% to 94.81%	88.89	67.20% to 98.03%	7.929
< 26.21	88.1	75.00% to 94.81%	83.33	60.78% to 94.16%	5.286
< 31.20	90.48	77.93% to 96.23%	83.33	60.78% to 94.16%	5.429

SARS-CoV-2 N					
CT	Sensitivity %	95% CI	Specificity %	95% CI	Likelihood ratio
< 14.87	73.81	58.93% to 84.70%	100	82.41% to 100.0%	
< 18.01	76.19	61.47% to 86.52%	100	82.41% to 100.0%	
< 21.09	76.19	61.47% to 86.52%	94.44	74.24% to 99.72%	13.71
< 21.40	76.19	61.47% to 86.52%	88.89	67.20% to 98.03%	6.857
< 28.85	78.57	64.06% to 88.29%	88.89	67.20% to 98.03%	7.071

Supplementary Table S5: Analysis of the sensitivity and specificity of different cycle threshold values from Receiver Operator Characteristic Curves for SARS-CoV-2 targets

The rows highlighted in green were selected as the adjusted cycle threshold (Cq value)

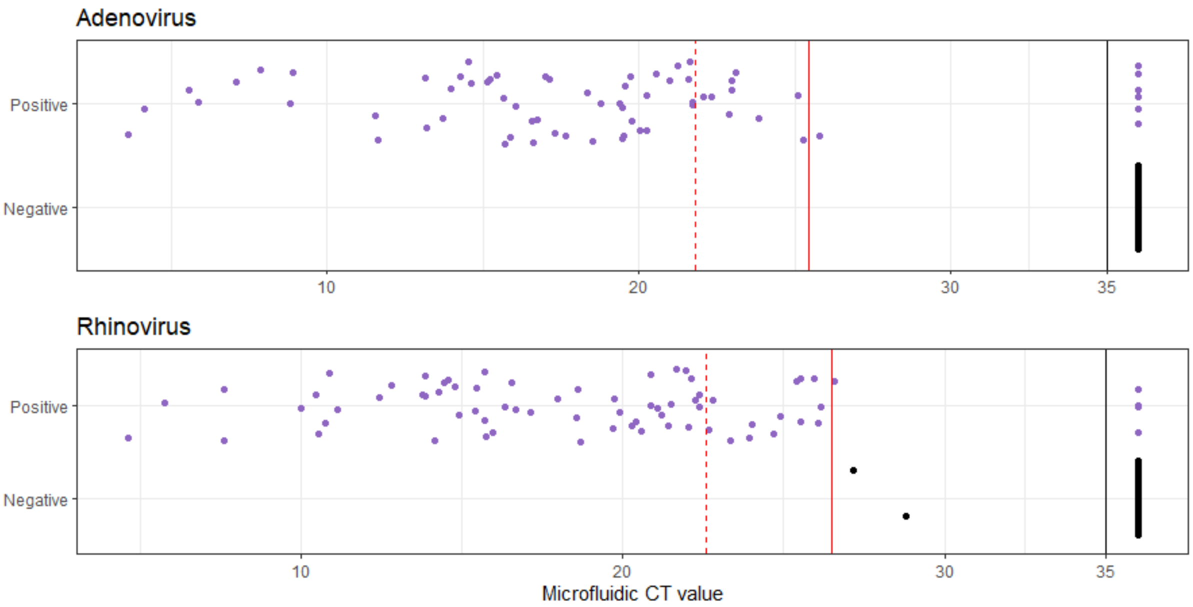

Supplementary Figure S3: Distribution of Cq values from duplicate testing of NASIMMUNE sample results on microfluidic testing. The original Cq cut off (red dashed line) and adjusted Cq cut off (red solid line) for each virus are shown. Samples were deemed positive if either of the two Cq values was under the adjusted Cq threshold. Samples over the maximum number of cycles performed (35) had “No Cq”.

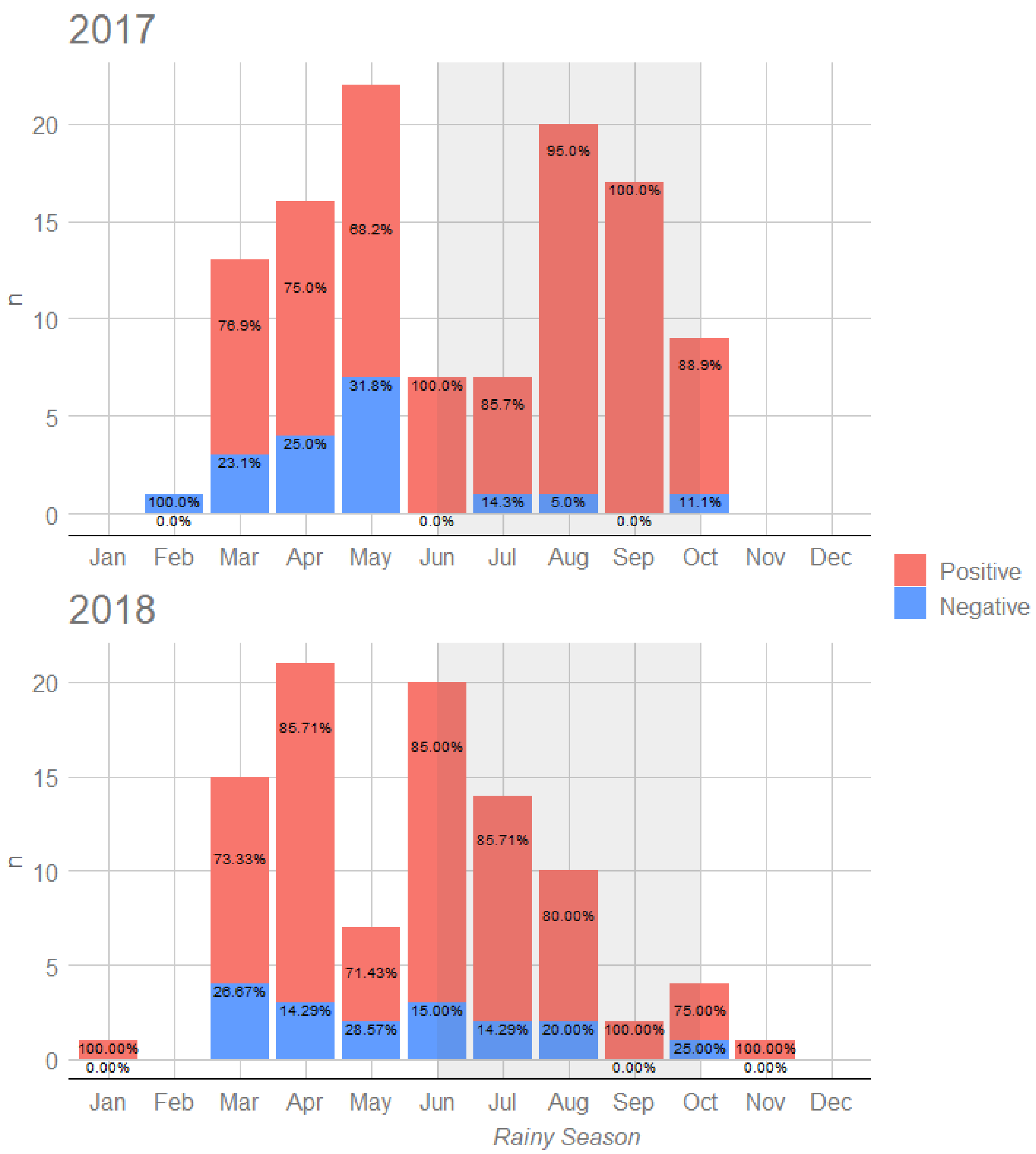

Supplementary Figure S4: Proportion of positive and negative results from upper respiratory tract swabs taken from children aged 24-59 months with influenza-like illness in the Gambia in 2017 and 2018. Only partial data are available for January and November, and no data for December, due to follow up windows in the parent study. Rainy season shaded in grey.

	Fever (≥37.5°C) at ILI visit	History of fever	Cough	Sore throat
Adenovirus	6/33 (18.1)	30/33 (90.9)	30/33 (90.9)	1/33 (3.0)
RSV A/B	4/8 (50.0)	8/8 (100)	8/8 (100)	0/8 (0.0)
Influenza A/B	9/18 (50.0)	18/18 (100)	16/18 (88.8)	0/18 (0.0)
Rhinovirus	5/35 (14.2)	31/35 (88.6)	31/35 (88.6)	1/35 (2.9)
HMPV A/B	6/19 (15.8)	18/19 (94.7)	19/19 (100)	0/19 (0.0)
Other viruses	4/27 (14.8)	24/27 (88.9)	25/27 (92.6)	0/27 (0.0)

Supplementary Table S6: Available Symptom data of children presenting with ILI with a subsequent positive result

HMPV: Human metapneumovirus, ILI: Influenza-like illness, RSV: Respiratory Syncytial Virus
